# Prevalence of genital *Chlamydia trachomatis* infection in the general population: a meta-analysis

**DOI:** 10.1186/s12879-020-05307-w

**Published:** 2020-08-08

**Authors:** Pengcheng Huai, Furong Li, Tongsheng Chu, Dianchang Liu, Jian Liu, Furen Zhang

**Affiliations:** 1 Shandong Provincial Hospital for Skin Disease, Shandong First Medical University, Jinan, China; 2 Shandong Provincial Institute of Dermatology and Venereology, Shandong Academy of Medical Sciences, Jinan, China

**Keywords:** *Chlamydia trachomatis*, Prevalence, General population, Meta-analysis

## Abstract

**Background:**

Estimating prevalence of Chlamydia trachomatis (CT) worldwide is necessary in designing control programs and allocating health resources. We performed a meta-analysis to calculate the prevalence of CT in the general population.

**Methods:**

The Pubmed and Embase databases were searched for eligible population-based studies from its inception through June 5, 2019. Q test and *I*^2^ statistic were used to calculate the heterogeneity between studies. Random effects models were used to pool the prevalence of CT. Meta regression was performed to explore the possible sources of heterogeneity. Publication bias was evaluated using a funnel plot and “trim and fill” method.

**Results:**

Twenty nine studies that reported prevalence of CT infection from 24 countries were identified, including a total population of 89,886 persons. The pooled prevalence of CT among the general population was 2.9% (95% CI, 2.4–3.5%), and females had a higher CT prevalence (3.1, 95% CI, 2.5–3.8%) than males (2.6, 95% CI, 2.0–3.2%) (*χ*^*2*^ = 10.38, *P* <  0.01). Prevalence of CT was highest in region of America (4.5, 95% CI, 3.1–5.9%), especially in Latin America (6.7, 95% CI, 5.0–8.4%), followed by females in region of Africa (3.8, 95% CI, 0.7–6.9%), while South-East Asia had a lowest CT prevalence 0.8% (95% CI, 0.3–1.3%).

**Conclusions:**

This study provided the updated prevalence of CT among general population worldwide. General population from Latin America, especially females, and women in Africa should be given priority by WHO when design and delivery CT control programs.

## Background

*Chlamydia trachomatis* (CT) causes the most prevalent bacterial sexual transmitted infection (STI) in the world [1]. Based on the 2018 global STI surveillance from World Health Organization (WHO), global estimation of new CT cases in 2016 was 127 million [2]. Chlamydia infections are asymptomatic in 61% of women and 68% of men, and as a consequence often go undiagnosed, untreated, resulting in onward transmission [3]. Left timely untreated, CT infection can lead to pelvic inflammatory disease (PID), infertility, ectopic pregnancy, and chronic pelvic pain in women [4], and urethritis, epididymitis in men [5]. Thus, early detection and treatment is necessary to reduce the burden of CT infections and associated sequelae.

Estimation of CT prevalence in the general population is essential in designing a specific control program [3]. Population-based studies have been conducted in several countries to estimate prevalence of CT infection [6–9]. However, sample size of many studies was too small to obtain the precise estimation considering the low CT prevalence in general population [10–12]. Meta-analysis can obtain large sample size and provide robust and reliable estimation of CT prevalence. In 2015, a meta-analysis conducted by WHO found the global prevalence of CT was 4.2% for women and 2.7% for men [13]. In the same year, Redmond et al. calculated the genital CT prevalence in Europe and non-European high income countries using meta-analysis [14]. In addition, Rowley et al. performed a systematic review to estimate global prevalence of CT in 2016, and the prevalence of CT for women and men was 3.8 and 2.7%, respectively [15]. However, the included population-based studies of these three meta-analyses were all published before 2016. Another 5 eligible studies were published in succession since 2016 [3, 16–19]. In addition, studies estimating prevalence of CT for selective populations such as high school students, military recruits, clinic based individuals were also included in these meta-analyses, which introduced potential heterogeneity for the pooled results [13, 20–22]. Thus, we preformed this meta-analysis to update global and regional prevalence estimates for CT infection in the general population and provide convincing information for design and delivery CT control programs.

## Methods

This study was conducted according to the meta-analysis of observational studies in epidemiology (MOOSE) guidelines [23].

### Search strategy

The Pubmed and Embase databases were searched for population-based studies reported prevalence of CT from its inception through June 5, 2019. The following search strategy was used to identify all relevant articles: “*Chlamydia trachomatis*” AND “prevalence” AND “population based study”. The languages of studies were limited to English and Chinese, and the subjects were defined as humans. The reference lists of included articles were also screened for further studies.

### Inclusion and exclusion criteria

Studies included in this meta-analysis met all the following inclusion criteria: (1) cross-sectional studies or baseline study of cohort studies; (2) reported data from a general population; (3) explicitly reported prevalence (or weighted prevalence) of CT infection, or number of infected cases; (4) used nucleic acid amplification test (NAAT) or cell culture for diagnostic testing. (5) Articles were published between January 1, 2000 and June 5, 2019. If multiple cross-sectional studies for the same subjects and areas were published, the most updated report was included. We excluded studies that (1) reported data from subsample or followed-up individuals; (2) reported on selective populations such as outpatients, pregnant women, military recruits, students, aborigines, or convicts; (3) used serologic test for CT diagnosis.

### Data extraction and quality assessment

Data were extracted from included studies on first author, year of publication, study area or country, gender of subjects, sample size, detection methods, prevalence (or weighted prevalence) of CT infection, number of infected cases. In addition, studies were divided into the following 6 subgroups based on WHO regions: African group, American group, South-East Asian group, European group, Eastern Mediterranean group and Western Pacific group. Quality assessment for included studies using the assessment criterion for cross-sectional/prevalence studies (11 items), which was recommended by the Agency for Healthcare Research and Quality (AHRQ) in U.S. [24]. We chose “yes”, “no” or “unclear” for each item based on the contents of the articles. Item answered with “yes” was scored 1, otherwise, it was scored with 0. Articles scored “8–11”, “4–7”, “0–3” were defined as high quality, medium quality and low quality articles, respectively. Data extraction and quality assessment were performed by 2 authors independently. Any disagreements were resolved through discussion.

### Statistical analysis

The pooled prevalence of CT infection and 95% confidence interval (CI) was calculated with STATA version 11 (StataCorp LP, College Station, TX, USA). Q test and *I*^2^ statistic were used to calculate the heterogeneity between studies. *I*^2^ describes the percentage of total variation because of between-study heterogeneity rather than chance, and it ranged from 0 to 100% [25]. Random (Dersimonian Laird method) effects models were used to calculate pooled prevalence of CT infection when heterogeneity was present (*I*^2^ > 50%), otherwise, fixed (Mantel Haenszel method) effects models were used [26]. Subgroup analysis was conducted according to WHO regions. Meta regression was performed to explore the possible sources of heterogeneity. Publication bias was evaluated using a funnel plot and “trim and fill” method. A *P* value < 0.05 was considered to be statistically significant.

## Results

### Search results and study characteristics

**Fig. 1:**
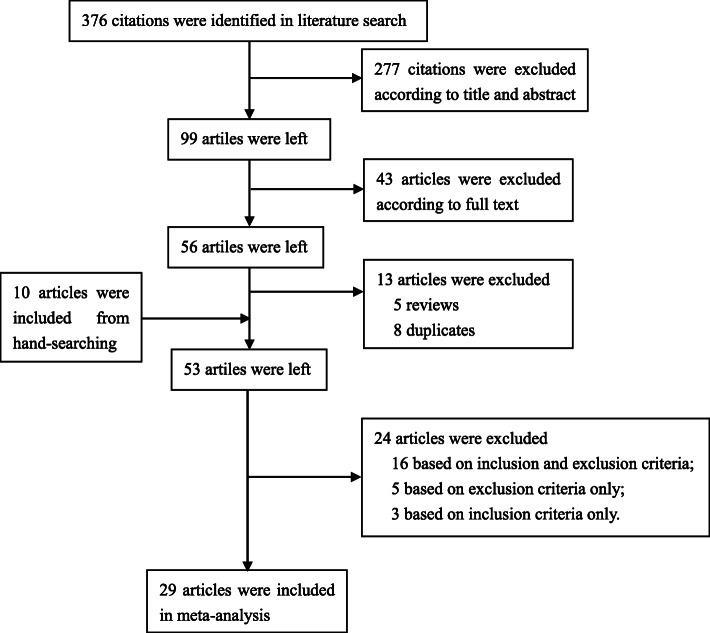
A flow chart of articles selection

In brief, a total of 29 eligible studies were identified and included into meta-analysis from 376 potentially relevant articles [3, 6–12, 16–19, 27–43]. A flow chart for inclusion and exclusion of articles was presented as Fig. 1. The included 29 studies, with a total population of 89,886 persons, reported prevalence of CT infection from 24 countries. Ten studies were conducted in Americas [6, 8, 27, 29, 33–35, 38, 40, 41], 9 in Europe [7, 10, 12, 28, 30, 31, 35–37], 7 in Western Pacific region [3, 9, 11, 18, 19, 32, 35], 4 in South-East Asia [16, 17, 35, 39] and 3 in Africa [35, 42, 43]. No eligible studies were retrieved from Eastern Mediterranean region. One study involved men only [43], 9 studies involved women only [11, 16–18, 32, 34, 35, 38, 41], 17 studies involved both men and women and reported sex-specific results [3, 6–10, 12, 19, 28–31, 33, 36, 37, 39, 42] and 2 studies involved both men and women but did not report sex-specific data [27, 40]. One article reported results from both cross-sectional study and baseline of cohort study [35], the other studies were all cross-sectional studies [3, 6–12, 16–19, 27–34, 36–43]. Based on assessment criterion, fifteen studies were defined as high quality articles [3, 6–9, 11, 12, 16, 27, 29, 31, 36, 40, 41, 43], 14 studies were medium quality articles [10, 17–19, 28, 30, 32–35, 37–39, 42], and no low quality articles were defined. The above characteristics of included studies were extracted in Table S1.

### Prevalence of CT infection

**Fig. 2:**
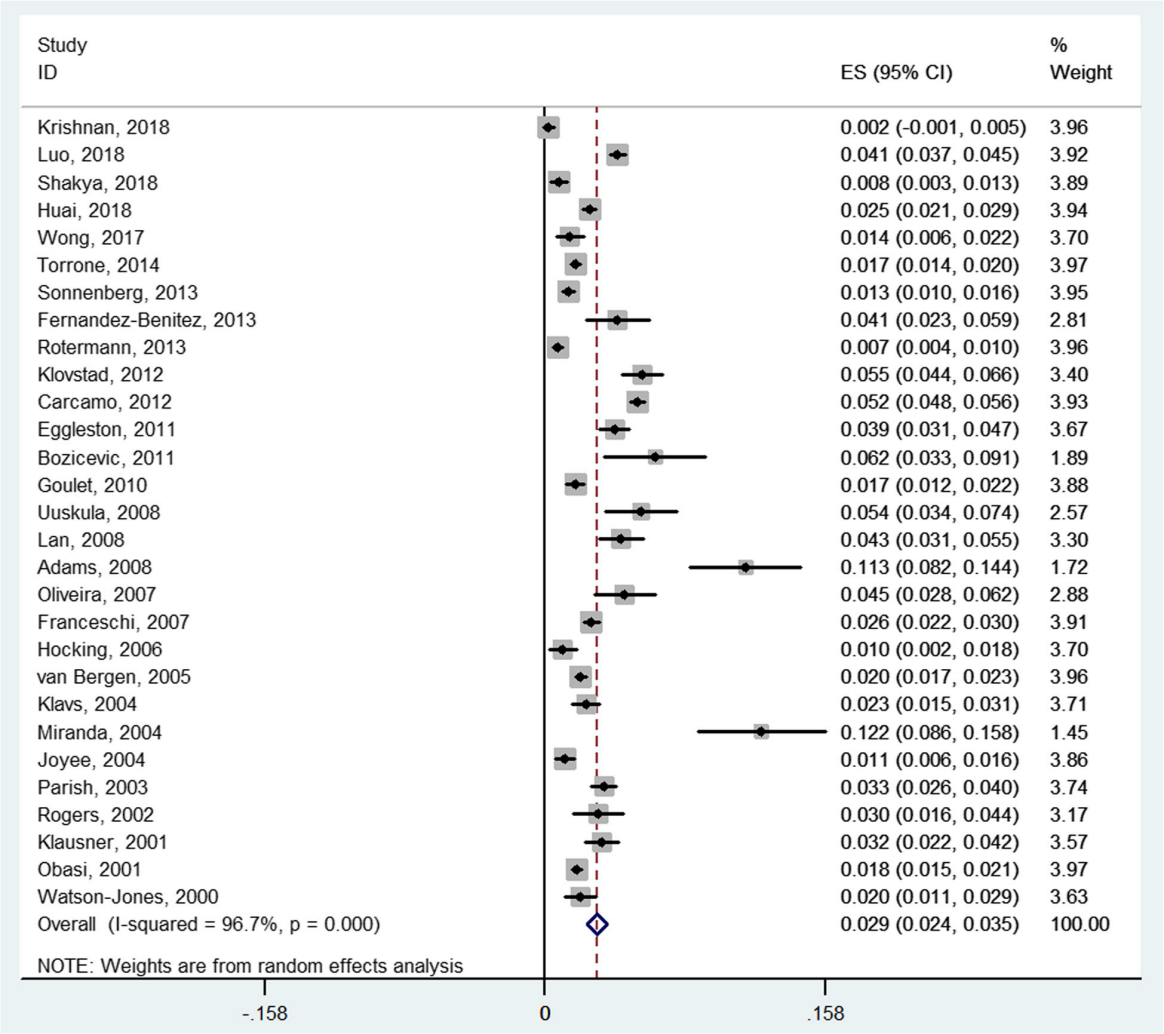
Random effects meta-analysis of the prevalence of CT infection among general population

Prevalence of CT infection ranged from 0.2 to 12.2% for the included studies. Q test indicated that there was significant heterogeneity between 29 studies (*P*_*Q*_ <  0.01; *I*^*2*^ = 96.7%), and a random effects model was used. The pooled prevalence of CT among the general population was 2.9% (95% CI, 2.4–3.5%) (Fig. 2). Prevalence of CT infection for men ranged from 1.0 to 12.1% for the 18 studies. A random effect model was used to pool the results because of the heterogeneity between studies (*P*_*Q*_ <  0.01; *I*^*2*^ = 92.2%). The pooled prevalence of CT infection for men was 2.6% (95% CI, 2.0–3.2%). Prevalence of CT infection for women ranged from 0.2 to 12.2% for the 26 studies. There was significant heterogeneity between studies (*P*_*Q*_ <  0.01; *I*^*2*^ = 96.0%), and a random effects model was used. The pooled prevalence of CT infection for women was 3.1% (95% CI, 2.5–3.8%). Result of Chi-square test indicated that prevalence of CT infection for women was significantly higher than that for men (*χ*^*2*^ = 10.38, *P* <  0.01).

### Subgroup analysis

**Fig. 3:**
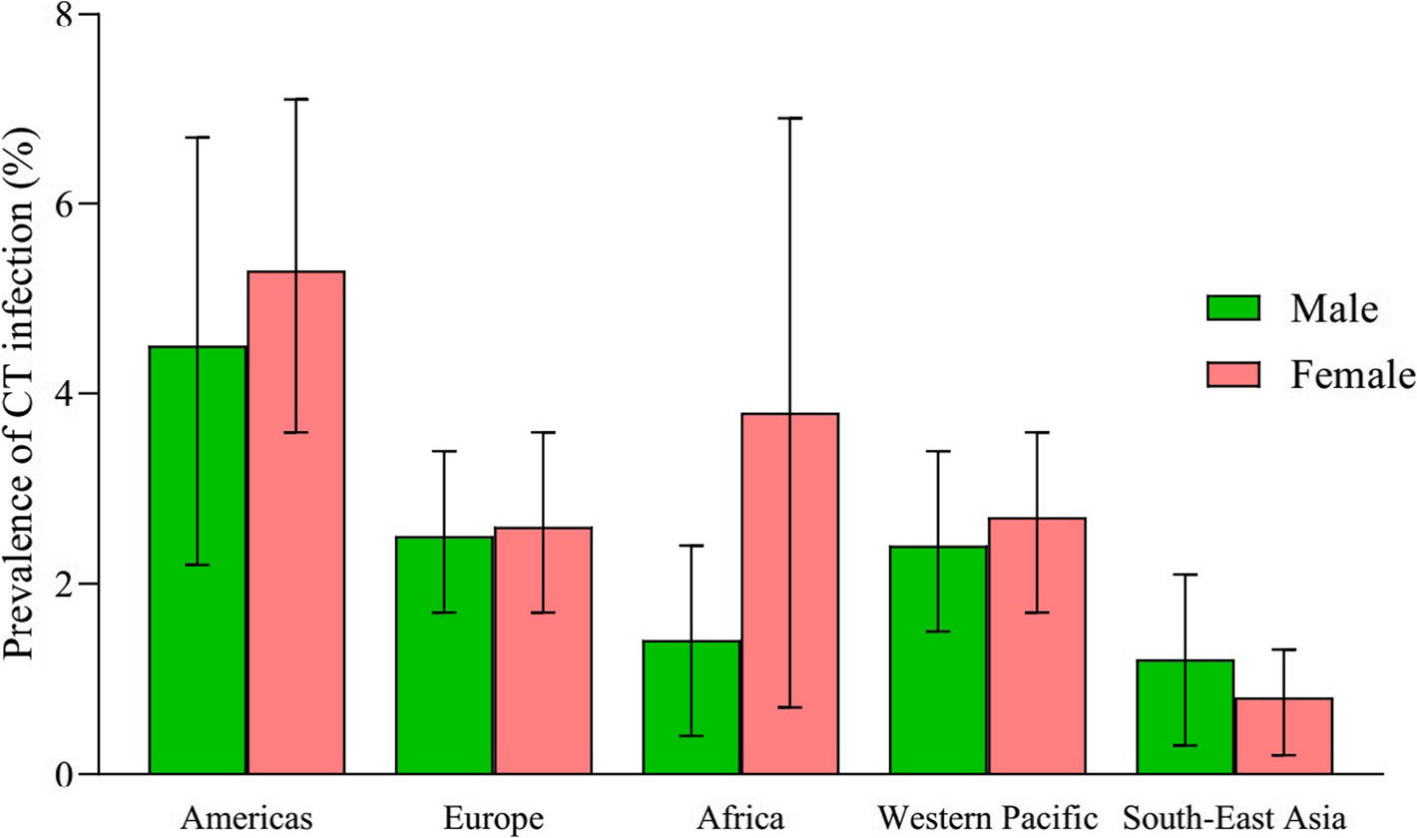
Pooled prevalence of CT infection among general population in 5 WHO regions

The pooled prevalence of CT infection in the general population in Americas was 4.5% (95% CI, 3.1–5.9%), which was calculated with a random effects model (*P*_*Q*_ <  0.01; *I*^*2*^ = 98.1%). Females (5.3, 95% CI, 3.6–7.1%) had a higher prevalence of CT than males (4.5, 95% CI, 2.2–6.7%) in the Americas (*χ*^*2*^ = 9.06, *P* < 0.01). Prevalence of CT in the population in Latin America (6.7, 95% CI, 5.0–8.4%) was higher than the prevalence in people from other countries in Americas (2.4, 95% CI, 1.4–3.4%) (*χ*^*2*^ = 333.2, *P* < 0.01). The pooled prevalence of CT in Europe, pooled with a random effects model (*P*_*Q*_ < 0.01; *I*^*2*^ = 94.2%), was 2.7% (95% CI, 1.9–3.6%). No significant difference was detected for the prevalence of CT between males and females in Europe (*χ*^*2*^ = 0.20, *P* = 0.68). The pooled prevalence of CT in Africa was 2.6% (95% CI, 1.4–3.9%), which was similar to that in the Western Pacific (2.6, 95% CI, 1.8–3.5%). Both subgroup meta-analyses were performed with random effects models. The prevalence of CT for females (3.8, 95% CI, 0.7–6.9%) was significantly higher than that for males (1.4, 95% CI, 0.4–2.4%) in Africa (*χ*^*2*^ = 63.44, *P* < 0.01). The difference of CT prevalence between males and females was not significant in the Western Pacific region (*χ*^*2*^ = 1.46, *P* = 0.23). Prevalence of CT in South-East Asia, pooled with a random effects model (*P*_*Q*_ < 0.01; *I*^*2*^ = 77.7%), was 0.8% (95% CI, 0.3–1.3%). The difference in prevalence of CT between males and females in South-East Asia was not significant (*χ*^*2*^ = 0.82, *P* = 0.47). Chi-square test indicated that the prevalence of CT infection among the 5 WHO regions was significantly different (*χ*^*2*^ = 413.5, *P* < 0.01). Detailed results of subgroup analysis were shown in Table 1 and Fig. 3.

**Table 1:** Subgroup analysis of prevalence of CT in the general population in 5 WHO regions

WHO regions	Male (%, 95 CI)	Female (%, 95 CI)	*χ*^*2*^	*P*	Total (%, 95 CI)
Americas	4.5 (2.2–6.7)	5.3 (3.6–7.1)	9.06	< 0.01	4.5 (3.1–5.9)
Europe	2.5 (1.7–3.4)	2.6 (1.7–3.6)	0.20	0.68	2.7 (1.9–3.6)
Africa	1.4 (0.4–2.4)	3.8 (0.7–6.9)	63.44	< 0.01	2.6 (1.4–3.9)
Western Pacific	2.4 (1.5–3.4)	2.7 (1.7–3.6)	1.46	0.23	2.6 (1.8–3.5)
South-East Asia	1.2 (0.3–2.1)	0.8 (0.2–1.3)	0.82	0.47	0.8 (0.3–1.3)
Total	2.6 (2.0–3.2)	3.1 (2.5–3.8)	10.38	< 0.01	2.9 (2.4–3.5)

*CT Chlamydia trachomatis*; *WHO* World Health Organization; *CI* Confidence interval

### Exploration of the heterogeneity source

Exploratory univariate meta-regression was conducted with the introduction of sample size, number of cases, year of publication, country, WHO regions and scores in quality assessment, response rate and national/subnational studies. The meta-regression coefficients, 95% CI and *P* value for these variables were − 8.68e^− 7^ (95% CI, − 3.65e^− 6^-1.92e^− 6^, *P* = 0.53), − 1.40e^− 6^ (95% CI, − 7.26e^− 5^-6.98e^− 5^, *P* = 0.97), − 6.21e^− 4^ (95% CI, − 2.32e^− 3^-1.08e^− 3^, *P* = 0.46), − 7.26e^− 4^ (95% CI, − 2.18e^− 3^-7.33e^− 4^, *P* = 0.32), − 3.85e^− 3^ (95% CI, − 1.08e^− 2^-3.11e^− 3^, *P* = 0.27), − 1.91e^− 3^ (95% CI, − 9.16e^− 3^-5.35e^− 3^, *P* = 0.59), − 1.84e^− 4^ (95% CI, − 3.04e^− 4^-6.72e^− 4^, *P* = 0.45), − 4.81e^− 3^ (95% CI, − 2.51e^− 2^-1.55e^− 2^, *P* = 0.63). Thus, no variable was identified to be the main source of heterogeneity in this meta-analysis of the whole 29 studies.

### Publication bias evaluation

The shape of the funnel plot was not symmetrical, which indicates the existence of publication bias for the included studies. Trim and fill method was used to adjust the pooled prevalence of CT infection. After adjustment, the pooled prevalence of CT infection among general population was 2.6% (95% CI, 2.0–3.1%).

## Discussion

This meta-analysis provided the updated estimation of prevalence of CT infection among the general population worldwide. This study included 29 studies with a total population of 89,886, which covered 24 countries from 5 WHO regions. The results of this study showed that prevalence of CT infection among general population was 2.9% (95% CI, 2.4–3.5%), and females had a higher CT prevalence than that for males. Prevalence of CT infection among the 5 WHO regions varied significantly. The general population in the Americas had the highest CT prevalence, followed by those in Europe, Africa, and the Western Pacific regions, while those in South-East Asia had the lowest CT prevalence.

The prevalence of CT infection in males was 2.6% (95% CI, 2.0–3.2%) in this study, which was very similar to the estimation (2.7, 95% uncertainty interval, UI, 1.9–3.7%) by Rowley et al. in 2016 [15]. However, the CT prevalence for females (3.1, 95% CI, 2.5–3.8%) was lower than the estimation (3.8, 95% UI, 3.3–4.5%) by Rowley [15], which may be due to the difference in inclusion criteria between two studies. Pregnant women, women at delivery, and women attending family planning clinics were recruited in the systematic review by Rowley, but those were excluded in our study. Pregnancy is a time of many changes in a woman’s life, including hormonal and physiological changes and reduced immune activity, making her vulnerable to infections [44]. Thus, females are more likely to acquire CT infections during pregnancy, which may result in the higher prevalence of CT estimated by Rowley et al. The pooled CT prevalence in Europe was in line with the meta-analysis by Redmond et al. in European Union/European Economic Area (EU/EEA) Member States, and the 95% CI were overlapped between two studies [14].

Our study found significant differences in the prevalence of CT infection between males and females in the general population. The higher CT prevalence in females may be due to several reasons. First, the anatomy of reproductive tract makes the females vulnerable to CT infection. Genital secretions and pathogen CT tend to pool in the vaginal vault, where they typically bathe the uterine cervix in the secretions, resulting in transmission of CT [45]. Second, cervical ectopy, which is a common physiological process in young women, was thought to increase risk of CT infection by exposing columnar epithelium to a potential infectious inoculum [46]. Third, Non-monogamous partnerships are more common among men than women [47]. As a consequence, men may transmit CT to multiple female partners, resulting in this increased CT prevalence. Considering cost-effective, females should be given priority when conduct CT control programs such as screening. In addition, contact tracing around an infected woman is also necessary to find out the men who might be responsible for several other infections. Therefore, CT screening, contact tracing, and cases treatment should be combined to prevent CT transmission.

The results of this study indicated regional variations of CT prevalence. Pooled CT prevalence was highest in the region of Americas and lowest in the South-East Asia. We further explored the heterogeneity between Latin America and U.S., Canada, and found a significant higher CT prevalence in population from Latin America. In U.S., all sexually active females ≤25 years of age were recommended to be screened for CT infections routinely since 2001 [48]. In Canada, sexually active young adult ≤25 years of age as well as pregnant women were recommended to be screened for CT infections [49]. Compared with Latin America, those CT control programs in U.S. and Canada may contribute to the lower CT prevalence. The results about spatial heterogeneity of CT prevalence provide information for WHO to design a CT control policy. The limited health resources, such as funding or human resources, should lean to the regions with high CT prevalence. Previous publications indicated that CT screening was cost effective for females at prevalence of 3.1–10% [50–52]. Thus, whether to conduct CT screening programs for females in Africa and Latin America should be evaluated by WHO. Regional variations may be associated with social, cultural and economic conditions, differences in control policy and gender inequality, but those need to be examined in further studies [53].

In this study, only general population were included into analysis, selective populations such as outpatients, military recruits or convicts were excluded from this study, which minimized the selection bias of this meta-analysis. There are also several limitations in this study. First, heterogeneity was found between studies through Q test. We used meta-regression to explore potential source of heterogeneity, but no variable was identified to be the main source of heterogeneity. Random effect models were used to pool the prevalence of CT, but the results may be not stable enough due to heterogeneity. Second, publication bias was identified for the included studies. Thus, trim and fill method was used to adjust the results and reduce the influence of publication bias. Third, only English and Chinese language articles were included in this meta-anlysis because the authors are fluent of these two languages. Eligible studies with other languages were not included, which may affect the results [26].

## Conclusions

In conclusion, our study provides the updated prevalence of CT among general population worldwide. Pooled prevalence of CT was calculated for males and females from different regions, and high-prevalence areas and populations were identified. Based on the results, the general population from Latin America, especially females, and women in Africa should be given priority by WHO when design and delivery CT control programs.

## Supplementary information

**Additional file 1 Table S1**. Characteristics of the 29 included studies in this meta-analysis.

## Data Availability

The dataset of this meta-analysis is included within the article’s additional file (Additional file 1).

## Abbreviations

AHRQ: Agency for healthcare research and quality
CI: Confidence interval
CT: Chlamydia trachomatis
EEA: European economic area
EU: European union
MOOSE: Meta-analysis of observational studies in epidemiology
NAAT: Nucleic acid amplification test
PID: Pelvic inflammatory disease
STI: Sexual transmitted infection
WHO: World health organization
